# Identification of the β-catenin/JNK/prothymosin-alpha axis as a novel target of sorafenib in hepatocellular carcinoma cells

**DOI:** 10.18632/oncotarget.5738

**Published:** 2015-10-22

**Authors:** Yi-Te Lin, Chuck C.-K. Chao

**Affiliations:** ^1^ Graduate Institute of Biomedical Sciences, College of Medicine, Chang Gung University, Taoyuan 33302, Taiwan, Republic of China; ^2^ Department of Biochemistry and Molecular Biology, College of Medicine, Chang Gung University, Taoyuan 33302, Taiwan, Republic of China

**Keywords:** apoptosis, hepatocellular carcinoma, β-catenin, prothymosin, sorafenib

## Abstract

Sorafenib is a kinase inhibitor used as anticancer drug against various human tumors, including advanced hepatocellular carcinoma (HCC). β-Catenin and prothymosin alpha (PTMA) are overexpressed in HCC and other tumors. Previous studies have shown that PTMA expression modulates the response of HCC cells to sorafenib. However, the underlying mechanism of PTMA activity in this context remains unclear. We show here that sorafenib inhibits both β-catenin and PTMA in a dose-dependent manner. Silencing β-catenin reduces PTMA level and sensitizes HCC cells to sorafenib. In contrast, ectopic expression of β-catenin induces PTMA expression and cell resistance to the drug. Sorafenib inhibits PTMA expression at the transcriptional level by inhibiting the β-catenin pathway. Nucleotide deletion analysis of the PTMA gene promoter reveals that a DNA segment lying 1,500–1,600 bp upstream of the PTMA transcription start site represents an AP-1-binding site that is critical for β-catenin modulation of gene transcription in response to sorafenib. In addition, chemical inhibitors that target JNK abrogate β-catenin/AP-1 binding to the endogenous PTMA gene and reduces PTMA transcription and protein expression. Silencing of β-catenin or c-Fos induces similar effects on gene regulation and these are reversed by ectopic expression of β-catenin. Mutations in the PTMA promoter at the predicted β-catenin/AP-1 binding site partly abrogate sorafenib's effects on PTMA transcription. These results indicate that PTMA is induced by the oncoprotein β-catenin and protects HCC cells against sorafenib-induced cell death. The β-catenin/JNK/PTMA axis may thus represent a novel target for chemotherapy against HCC.

## INTRODUCTION

Hepatocellular carcinoma (HCC) is a complex liver disease associated with high mortality and prevalency worldwide, including in Southeast Asia and Taiwan [1]. HCC patients often respond poorly to current clinical therapies, including chemotherapy. Poor prognosis and high mortality rates are attributed in part to the difficulty in obtaining a diagnosis at an early stage. Alterations, up-regulations and mutations in genes and cellular signaling pathways have been shown to promote hepatocarcinogenesis, even leading to protection of cancer cells against clinical treatment. Our previous studies showed that the anti-apoptotic protein HURP (hepatoma upregulated protein) and PTMA (prothymosin-α) [2, 3], which are upregulated in human HCC [4, 5], represent important targets of sorafenib in HCC cells cultured *in vitro* [3, 6].

β-Catenin is involved in the development of many tumors, including HCC. Previous studies have shown that the β-catenin protein can be modified by mutations, by inactivated APC (adenomatous polyposis coli) or the Wnt signaling pathway. These modifications induce β-catenin accumulation in the nucleus and up-regulation of factors that act downstream of β-catenin, such as TCF (T-cell factor) family-associated genes (c-Myc and cyclin D1), leading to initiation of carcinogenesis and cancer progression [7–9]. A previous study also revealed that β-catenin mutations are observed in different transgenic mouse HCC cell lines obtained by overexpression of the oncogenes *c-myc* or H-*ras* [10]. β-Catenin containing activating mutations is prevalent in human HCC patients and cancer cell lines [7, 11–13], with a frequency of around 16% [13]. β-Catenin activation by extracellular Wnt family signals is also likely to promote cancer invasion and resistance to chemotherapy [14]. Therefore, targeting β-catenin may represent an attractive option for the development of novel clinical therapies [15].

Sorafenib is a standard therapy for advanced HCC but provides limited survival benefits. This drug represents an anti-angiogenic multiple kinase inhibitor that induces cell death by targeting the RAF/MEK/ERK pathway, as well as VEGFR (vascular endothelial growth factor receptor), PDGFR (platelet derived growth factor receptor)-β, KIT, FLT-3, RET, and Wnt/β-catenin [16–18]. Some studies have shown that inhibition of β-catenin by sorafenib is observed in HCC cell lines, liver cancer stem cells, and mice bearing HepG2 cell-derived tumors [19–21]. A recent study also shows that a combination of sorafenib and β-catenin inhibitors produces synergistic effects in hepatoma cells [22], suggesting that this strategy may represent a potential novel anti-cancer treatment. However, the detailed molecular mechanism of β-catenin inhibition in sorafenib-induced cell death remains unclear.

In a previous report, PTMA expression and localization was shown to vary during hepatocyte proliferation and apoptosis in rat hepatocytes [23]. In addition, PTMA was found to be highly expressed in human HCC [5]. However, the mechanism underlying regulation of PTMA expression and the possibility that this protein might produce anti-apoptotic effects in sorafenib-treated HCC cells have not been studied. High levels of PTMA and c-Myc co-expression were detected in various human tumors, including HCC [24–26]. c-Myc was initially found to upregulate PTMA transcription [27], and c-Myc-binding sites were identified in the proximal promoter and first intron of the PTMA gene [28–30]. We recently found that PTMA may play a role in the development of human HCC as c-Myc-binding sites were detected in the proximal promoter of PTMA [6].

In the present study, we show that PTMA is upregulated by β-catenin and that PTMA upregulation is inversely correlated with sorafenib sensitivity in HCC cells such as Mahlavu and J7. Notably, we identify a sorafenib-responsive element in the PTMA promoter, and demonstrate that sorafenib inhibits PTMA expression at the transcriptional level through inactivation of the β-catenin/JNK pathway.

## RESULTS

### Sorafenib-induced apoptosis is associated with down-regulation of β-catenin and anti-apoptotic proteins

**Figure 1:**
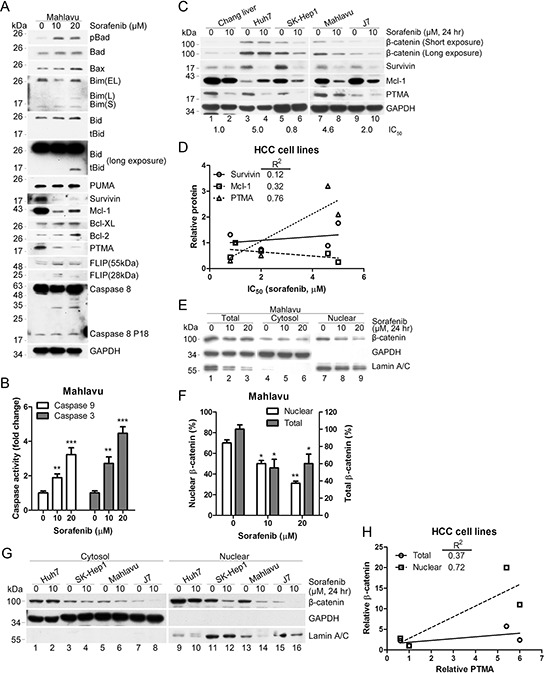
Sorafenib inhibits β-catenin levels and induces mitochondrial apoptotic pathways **A.** PTMA and other antiapoptotic proteins are down-regulated by sorafenib. PTMA, Mcl-1 and survivin protein levels were severely reduced in HCC Mahlavu cells exposed to sorafenib. **B.** Activation of caspase-9 and caspase-3 in sorafenib-treated Mahlavu cells. The cells were treated with sorafenib at the indicated concentrations for 24 hrs and caspase activities in cell lysates were measured using a colorimetric assay. **C.** Co-inhibition of β-catenin and PTMA by sorafenib in HCC cells. Cell lines indicated on top were treated or not with 10 μM sorafenib for 24 hrs and processed for immuno-blotting. IC_50_ values (the concentration of sorafenib that inhibits 50% of cell growth) for each cell line are indicated below the panels. **D.** Relative PTMA level correlated with the IC_50_ values of HCC cell lines. Correlation between survivin/Mcl-1 and IC_50_ values is also shown. Correlation index, R^2^ = 0.76, for PTMA was greater than for the two other proteins. **E.** Reduction of nuclear β-catenin by sorafenib. Mahlavu cells were treated with the indicated concentration of sorafenib for 24 hrs and cell extracts were prepared. **F.** Statistical plot of experiments shown in (E) (G) Reduction of nuclear β-catenin by sorafenib in HCC cell lines. **H.** Relative nuclear β-catenin level correlated with relative PTMA level. Plot of experiments shown in (C and G). Results of three experiments are expressed as mean ± SD. *P < 0.05, **P < 0.01, ***P < 0.001. Fifty μg of proteins from each sample were processed for immuno-blotting.

To examine how sorafenib induces apoptosis in HCC cell lines, we monitored the proteins involved in the intrinsic and mitochondrial apoptosis pathways, including pro-apoptotic proteins (Bad, Bax, Bim, Bid, and PUMA) and anti-apoptotic proteins (survivin, Mcl-1, Bcl-XL, Bcl-2, and PTMA). While Bax and Bid protein levels were slightly upregulated by sorafenib (at 20 μM but not 10 μM) in Mahlavu cells, the anti-apoptotic proteins survivin, Mcl-1 and PTMA were considerably down-regulated by the drug (Figure 1A). Furthermore, the extrinsic apoptosis pathway, which is associated with activation of cell surface death receptor, was activated to a low degree by sorafenib, as revealed by slight fragmentation of the anti-apoptotic protein FLIP and cleavage of caspase-8. Sorafenib also activated caspase-9 and caspase-3 in a dose-dependent manner in Mahlavu cells (Figure 1B). These results suggest that sorafenib may kill HCC cells by activating the mitochondrial apoptosis pathway.

Previous studies have shown that β-catenin may represent a target of sorafenib and a potential transcription factor that promotes chemoresistance through regulation of anti-apoptotic signals, such as survivin and Mcl-1 [31–33]. We observed that a lethal dose of sorafenib down-regulated β-catenin, survivin, Mcl-1, and PTMA in non-cancerous Chang liver and HCC cells (Huh7, SK-Hep1, Mahlavu, and J7) (Figure 1C, note that Mcl-1 increased in Huh7 cells). IC_50_ values produced by sorafenib varied in the HCC cell lines tested (the values are shown at the bottom of each Western blots in Figure 1C). Among the anti-apoptotic proteins tested, PTMA protein levels positively correlated with IC_50_ values (R^2^ = 0.76, Figure 1C and 1D; see also ref. [6]). Decreased protein levels and nuclear translocation of β-catenin were detected following sorafenib treatment (Figure 1E and 1F). While β-catenin levels in whole cell extracts did not show a high degree of correlation with PTMA, β-catenin in nuclear extracts showed excellent correlation with PTMA (Figure 1G, 1H). Co-reduction of β-catenin and PTMA was observed in the HCC cells examined (except Huh7, in which no change of β-catenin levels was observed); yet, a substantial reduction of PTMA levels was noted following sorafenib treatment. Nuclear β-catenin levels (or β-catenin activity) and PTMA levels were inversely correlated with sorafenib sensitivity in most HCC cell lines. These results suggest that sorafenib-induced apoptosis in HCC cells may be attributed to down-regulation of β-catenin and anti-apoptotic proteins and to activation of the caspase-9-dependent apoptosis pathway. Notably, total and nuclear β-catenin levels were not reduced by ERK inhibition in HCC cells (Supplementary Figure S1), an observation which contrasts with a previous study showing that the RAF1-p-ERK-β-catenin pathway is required for expansion of breast cancer-initiating cells and enhanced cancer malignancy [19].

### Silencing of β-catenin down-regulates PTMA and enhances sorafenib-induced apoptosis

**Figure 2:**
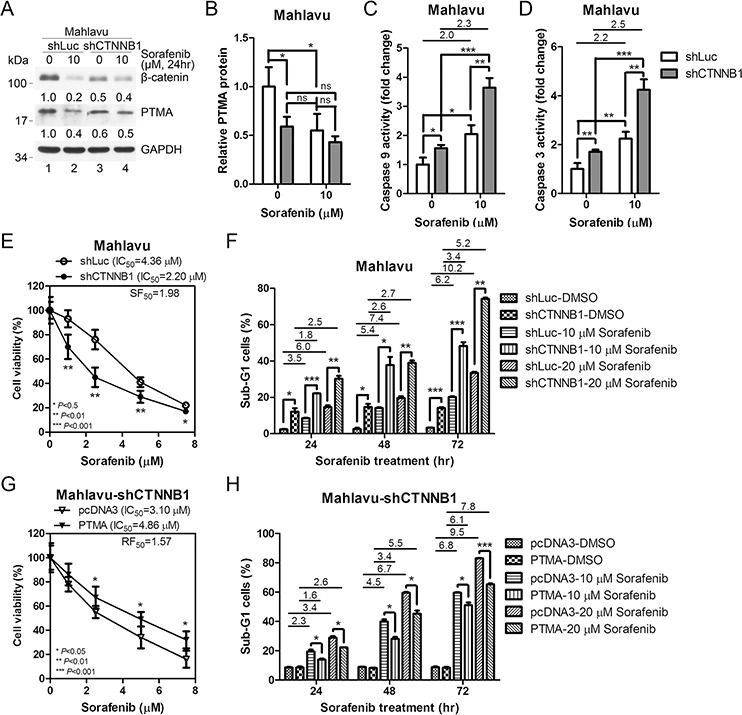

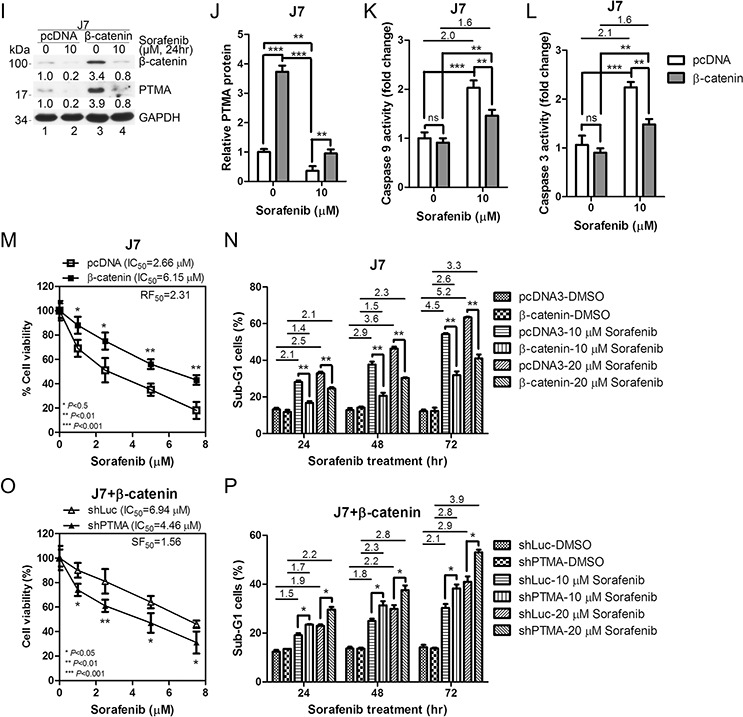
β-catenin silencing down-regulates PTMA protein level and potentiates sorafenib-induced cell death in HCC cells **A.** Down-regulation of PTMA protein level by β-catenin silencing (shCTNNB1) and sorafenib. Silencing caused inhibition of β-catenin by 50%. Fold change, relative to the control (first lane), is indicated. **B.** Quantification of PTMA protein level of (A) The relative protein level indicated (each lane was first normalized to GADPH) was calculated against the shLuc control. Increased activation of caspase-9 **C.** and caspase-3 **D.** by sorafenib following β-catenin silencing in Mahlavu cells. **E.** Sensitization of Mahlavu cells to sorafenib following β-catenin silencing. Cell sensitivity was assessed using the MTT assay. IC_50_ and sensitization factor (SF_50_) are indicated. SF_50_ was calculated by dividing the IC_50_ of control shLuc cells by that of shCTNNB1 cells. **F.** Enhancement of sorafenibinduced sub-G1 cells following β-catenin silencing. **G.** Ectopic expression of PTMA rescues sorafenib sensitivity in shCTNNB1-expressing Mahlavu cells. **H.** Reduction of sorafenib-induced sub-G1 cells following PTMA overexpression in shCTNNB1-expressing Mahlavu cells. **I.** Enhancement of PTMA following overexpression of β-catenin in J7 cells. **J.** Quantification of PTMA protein level of (I) Increased activation of caspase-9 **K.** and caspase-3 **L.** by sorafenib following β-catenin silencing in Mahlavu cells. **M.** Ectopic expression of β-catenin protects J7 cells against sorafenib. **N.** Reduction of sorafenib-induced sub-G1 cells following β-catenin overexpression. **O.** Sensitization of β-catenin-expressing J7 cells to sorafenib following PTMA silencing. **P.** Enhancement of sorafenib-induced sub-G1 cells in β-catenin-overexpressing J7 cells by PTMA silencing. Fold change between treatments is also indicated. Results of three experiments are expressed as mean ± SD. *P < 0.05, **P < 0.01, and ***P < 0.001.

Previous work showed that β-catenin silencing decreases PTMA mRNA levels in glioma cells [34], but the detailed mechanism underlying PTMA regulation remains unknown. To assess the role of β-catenin in sorafenib-induced apoptosis, we silenced the β-catenin gene (CTNNB1) using shRNA (shCTNNB1) in Mahlavu cells which express high levels of PTMA (Figure 1C). Our results showed that CTNNB1 silencing induces a decrease of PTMA protein levels in sorafenib-treated cells (Figure 2A and 2B). While caspase-9 and caspase-3 activity increased following sorafenib treatment in Mahlavu cells, β-catenin silencing induced the activity of the two caspases in untreated cells (Figure 2C and 2D). We also observed that shCTNNB1 sensitized HCC cells to sorafenib compared to shLuc control (Figure 2E). Sensitization was quantified using a sensitization factor (SF_50_), which was calculated by dividing the IC_50_ of control cells by that of shCTNNB1 cells (Figure 2E, SF_50_ = 1.98). Sorafenib induced the accumulation of sub-G1 cells in a dose- and time-dependent manner, and silencing of CTNNB1 further enhanced the effects of sorafenib on sub-G1 cell levels (Figure 2F). In contrast, ectopic expression of PTMA partly rescued shCTNNB1-induced sensitization to the drug as assessed by cell viability assay (with a resistance factor, RF_50_ = 1.57) or sub-G1 cell analysis (Figure 2G and 2H). These results indicate that the sensitization effect of β-catenin silencing to sorafenib involves PTMA.

### Ectopic expression of β-catenin up-regulates PTMA and reduces sorafenib-induced apoptosis

To verify the role of β-catenin and PTMA in regulating sorafenib-induced apoptosis, we overexpressed β-catenin in HCC J7 cells which express low levels of PTMA (Figure 1C). PTMA protein level increased following ectopic expression of β-catenin (Figure 2I and 2J). While β-catenin level was overexpressed 3.4 fold, PTMA protein levels doubled in the absence of sorafenib (Figure 2I, compare lanes 1 and 3; Figure 2J). Similar to the results obtained for Mahlavu cells, sorafenib reduced β-catenin levels by 80% in J7 cells expressing the control pcDNA3 vector (Fig, 2I, lanes 1 and 2). PTMA protein level was upregulated 3.9 fold following overexpression of β-catenin in the presence of sorafenib (Figure 2I, compare lanes 2 and 4; Figure 2J). These results suggest that β-catenin may be required, but not sufficient, for PTMA up-regulation. Sorafenib-induced caspase-9 and caspase-3 activities, which both increased 2 fold, were partially inhibited by β-catenin overexpression (Figure 2K and 2L). Sorafenib-induced inhibition of cell viability and sorafenib-induced sub-G1 cell accumulation were both significantly reversed by β-catenin overexpression (Figure 2M and 2N). Furthermore, induction of cell resistance to sorafenib by β-catenin overexpression was significantly reversed by PTMA silencing (Figure 2O and 2P). These results indicate that ectopic expression of β-catenin up-regulates PTMA and induces resistance to sorafenib-induced apoptosis. In addition, these findings suggest that PTMA mediates the effects of β-catenin on HCC cells.

### β-catenin silencing down-regulates PTMA mRNA level and PTMA promoter activity

**Figure 3:**
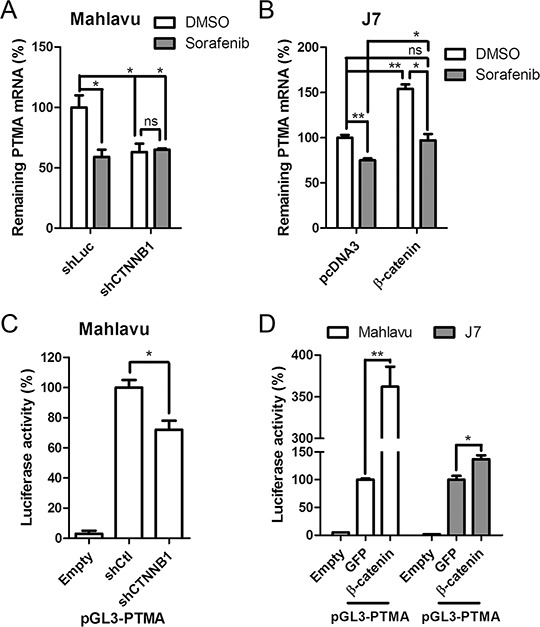
Sorafenib and β-catenin silencing attenuates PTMA expression through inhibition of mRNA expression **A.** Suppression of PTMA mRNA expression by sorafenib or β-catenin silencing in Mahlavu cells. PTMA mRNA levels were evaluated by qPCR. mRNA levels were compared to DMSO control. **B.** Restoration of PTMA mRNA expression by β-catenin overexpression in J7 cells. **C.** Reduction of PTMA promoter activity by β-catenin silencing. Relative luciferase activity of the PTMA promoter (pGL3-PTMA, see “Materials and Methods”) in β-catenin silencing (shCTNNB1) Mahlavu cells was divided by the luciferase activity of shControl cells, by setting shControl cells as 100%. **D.** Enhancement of PTMA promoter activity by β-catenin overexpression. Relative luciferase activity of the PTMA promoter in β-catenin overexpressing Mahlavu or J7 cells was divided by the luciferase activity of GFP overexpressing control cells, by setting GFP cells as 100%. Results of three experiments are expressed as mean ± SD. *P < 0.05, **P < 0.01; ns, not significant.

We have recently demonstrated that sorafenib down-regulates PTMA at the transcription level through c-Myc and that this phenomenon plays a significant role in regulating HCC cell sensitivity to the drug [6]. Here, we found that β-catenin plays an important role in up-regulating PTMA transcription. To assess the role of β-catenin in the regulation of PTMA expression, we monitored PTMA mRNA levels in HCC cells following silencing or overexpression of β-catenin. Sorafenib treatment (10 μM) or β-catenin silencing (shCTNNB1) reduced PTMA mRNA levels by almost 50% in Mahlavu cells (Figure 3A). PTMA mRNA levels were not further reduced in cells expressing shCTNNB1 (Figure 3A). Similar results were obtained when PTMA protein levels were monitored (Figure 2B). Sorafenib also reduced PTMA mRNA levels in J7 cells (Figure 3B). In addition, we detected a significant increase of PTMA mRNA levels following ectopic expression of β-catenin in J7 cells (Figure 3B). The effects produced by sorafenib or β-catenin on PTMA mRNA levels were similar to that observed earlier for the PTMA protein in J7 cells (Figure 2J). Notably, inhibition of PTMA mRNA and protein levels by sorafenib could be rescued by ectopic expression of β-catenin. While silencing of β-catenin (shCTNNB1) down-regulated the transactivation activity of the isolated PTMA promoter pGL3-PTMA by 30% (*P* < 0.05) [6] compared to control Mahlavu cells (shCtl), ectopic expression of β-catenin enhanced the promoter activity by over 3.5 fold (*P* < 0.01) compared to control (GFP) (Figure 3C and 3D). Up-regulation of PTMA promoter by β-catenin overexpression was also observed in J7 cells (*P* < 0.05) (Figure 3D, 1.5 fold). These results indicate that down-regulation of PTMA transcription by sorafenib is mediated by β-catenin. Our findings also suggest that the control of PTMA expression by sorafenib occurs mainly via the action of β-catenin on transcription.

### Identification of a putative β-catenin-responsive element in the PTMA gene promoter

**Figure 4:**
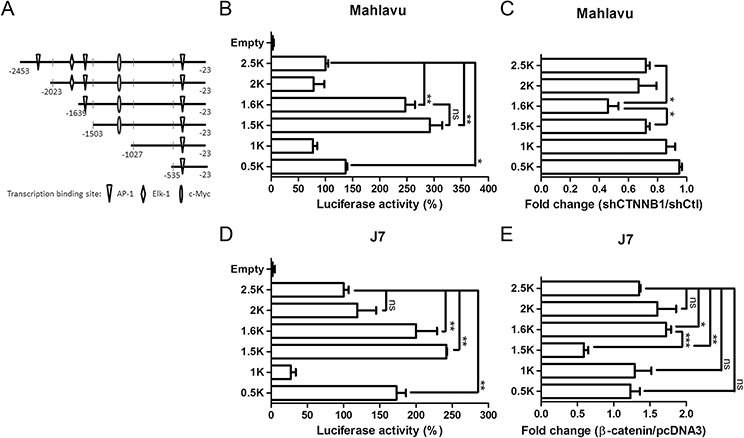
Determination of β-catenin responsive element in the PTMA promoter **A.** Schematic representation of the PTMA promoter. Full-length PTMA promoter contains regions of 2.5-kbp upstream of the transcription start site. Mutants are truncated from the 5′-end with nucleotides upstream of the transcription start site indicated. Transcription factor binding sites are also indicated. **B.** Relative luciferase activities of each promoter in Mahlavu cells. Luciferase activity of the full-length PTMA promoter (2.5 kb) was taken as 100%. Other plasmids corresponding to each mutant are indicated. **C.** Fold change of PTMA promoter activity following β-catenin silencing in Mahlavu cells. Fold change was calculated by dividing the luciferase activity of each promoter in shCTNNB1-treated cells by the luciferase activity of shCtl control cells. **D.** Relative luciferase activities of each promoter in J7 cells. **E.** Fold change of PTMA promoter activity following β-catenin silencing in J7 cells. Results of three experiments are expressed as mean ± SD. *P < 0.05, **P < 0.01, and ***P < 0.001; ns, not significant.

**Figure 5:**
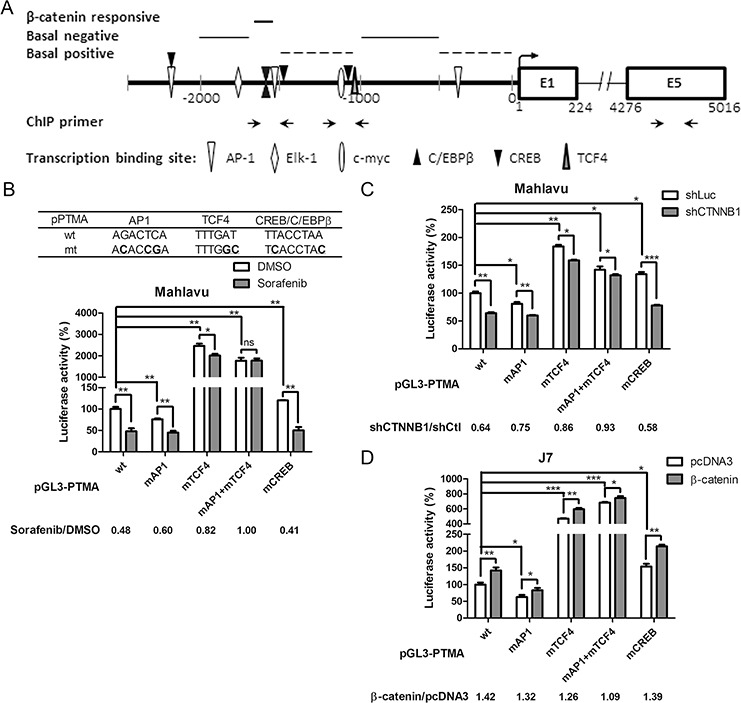
β-catenin responsive element overlapped with AP-1 binding element of the PTMA promoter **A.** Schematic representation of the PTMA promoter and flanking regions. The scheme summarizes the results of Figure 4. Positions of nucleotides relative to the transcription start site and predicted regions for β-catenin responsive, basal positive and negative regulation elements are indicated. Transcription factor binding sites are also indicated. **B.** Partial loss of response to sorafenib in mutant PTMA promoter. Partial sequences of wild-type (wt) and mutated (mt) plasmids are listed on top. Luciferase activity of the PTMA promoter with mutated site for AP-1 (mAP-1), TCF4 (mTCF4), both (mAP1+mTCF4), or CREB (mCREB) was also measured, taking the wild-type (wt) PTMA promoter as 100%. Fold change was calculated by dividing the luciferase activity of each promoter in DMSO control cells by the luciferase activity of sorafenib-treated cells (shown at the bottom). **C.** Partial loss of response to silencing β-catenin in mutant PTMA promoter in Mahlavu cells. Luciferase activity of wild-type PTMA promoter was inhibited by silencing β-catenin (shCTNNB1). Fold change was calculated by dividing the luciferase activity of each promoter in shCtl control cells by the luciferase activity of shCTNNB1-treated cells (shown at the bottom). Other symbols are the same as in (B). **D.** Partial loss of response to β-catenin overexpression in mutant PTMA promoter in J7 cells. Luciferase activity of wild-type PTMA promoter was increased to 1.42 fold by overexpressing β-catenin. Fold change was calculated by dividing the luciferase activity of each promoter in β-catenin plasmid-treated cells by the luciferase activity of pcDNA3-treated cells (shown at the bottom). Averaged results of three experiments are shown. n.s., not significant; *, *P* < 0.05; **, *P* < 0.01, ***, *P* < 0.005.

To identify putative β-catenin-responsive elements in the PTMA promoter, we performed a series of deletion from the 5′-end of the pGL-PTMA promoter (2.5 kb) in Mahlavu cells (Figure 4A). Basal transcription analysis revealed negatively- and positively-regulated elements in the −2023/−1639 and −1503/−1027 segments, respectively (Figure 4B). While activity of the 1.6 kb promoter was significantly down-regulated by β-catenin silencing (shCTNNB1) compared to shCtl control, the 1.5 kb promoter showed activity similar to that of the 2.5 kb promoter (Figure 4C), suggesting that the −1639/−1503 segment may contain a putative β-catenin-responsive element (Figure 5A). Basal expression pattern of these plasmid truncates, with similar negatively- and positively-regulated elements, was also found in J7 cells (Figure 4D). While the 1.6 kb truncated plasmid displayed significant activation following β-catenin overexpression, the 1.5 kb plasmid showed significant reduction in transactivity (Figure 4E). These results suggest that the −1639/–1503 segment of the PTMA promoter is critical for the response to β-catenin.

### Characterization of the β-catenin-responsive element in PTMA promoter activation: overlap with AP-1 and TCF4 enhancers

Transcription activity and critical segments for basal and regulated expression of the 2.5-Kb PTMA promoter was identified (see above). The PTMA promoter region contains a few major transcription factor sites (Figure 5A). The β-catenin-responsive region appears to overlap with AP-1, C/EBPβ and CREB elements. β-catenin always regulates gene expression by interacting with other transcription factors, such as TCF4 [35]. To verify the involvement of β-catenin and other transcription factors in activating the PTMA promoter, we created mutations in these predicted transcription factor-binding sites in the PTMA promoter, including for mAP1, mCREB, and mTCF4 (Figure 5B). While wild-type promoter activity was suppressed 0.48 fold by sorafenib in Mahlavu cells, the activity of the AP1, TCF4 and combined AP1/TCF4 mutant promoters was suppressed 0.60 fold, 0.82 fold and 1.00 fold, respectively (Figure 5B). On the other hand, the CREB mutant promoter activity displayed the same level of sorafenib-induced suppression as the wild-type promoter. These results indicate that mutation in AP1 and TCF4 binding sequences reduced the suppression induced by the drug. Suppression of promoter activity of mAP1, mTCF4 and combined mAP1/mTCF4, but not mCREB, was significantly reduced by β-catenin silencing (Figure 5C). Furthermore, potentiation of promoter activity by β-catenin overexpression was reduced for mAP1, mTCF4 and combined mAP1/mTCF4, but not for mCREB, compared to wild-type promoter in J7 cells (Figure 5D). These results indicate that AP1 and TCF4 binding sites are involved in the regulation of PTMA gene expression by sorafenib and β-catenin. Early studies have identified physical interactions between β-catenin and AP1 in stimulating TCF4-dependent gene expression [36, 37]. The promoter with double mutations at the AP1 and TCF4 binding sites displayed greater reduction of activity following sorafenib treatment than the single mutant AP1 or TCF4, suggesting that AP1 and TCF4 enhancers may overlap with β-catenin on the PTMA gene.

### Characterization of β-catenin-responsive element in the PTMA gene using ChIP assay: sorafenib-induced inhibition of β-catenin, AP-1 and TCF4 binding separately or in combination

**Figure 6:**
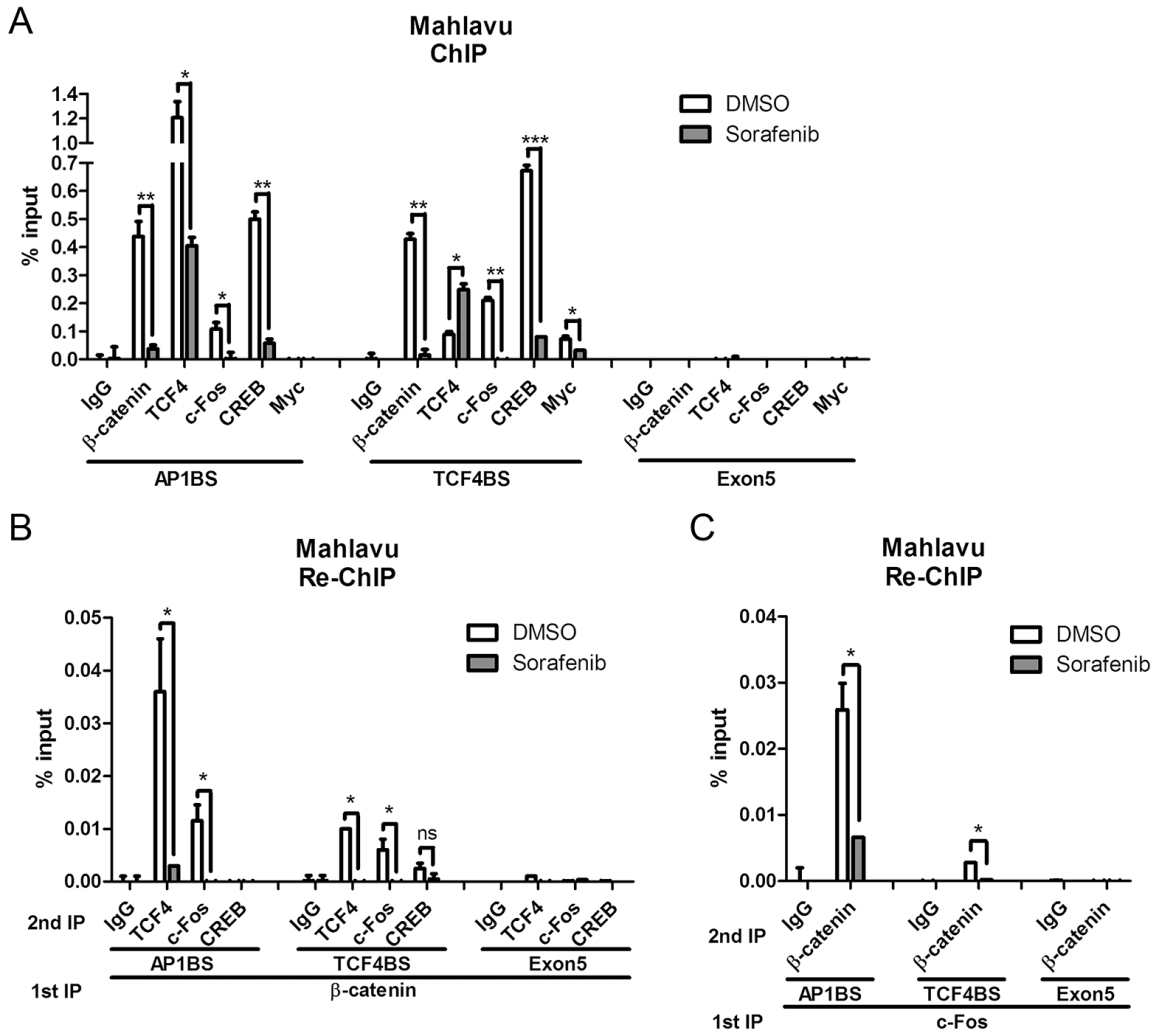
Inhibition of β-catenin and associated c-Fos binding to PTMA promoter by sorafenib using ChIP assay in Mahlavu cells Quantified average binding activities of the indicated factors to site of AP-1 (AP1BS), TCF4 (TCF4BS) or exon 5 (as shown in % input) by qPCR were calculated (see “Materials and Methods”). **A.** Inhibition of β-catenin and other transcription factors binding to the PTMA promoter by sorafenib using single ChIP assay. **B.** Inhibition of β-catenin and associated transcription factors binding to the PTMA promoter by sorafenib using re-ChIP assay. Cell protein extracts were first immunoprecipitated (IP) for β-catenin, followed by a second IP for the indicated transcription factors or IgG. Quantified average binding activities by qPCR were calculated. **C.** Inhibition of c-Fos and associated β-catenin binding to the PTMA promoter by sorafenib using re-ChIP assay. Cell protein extracts were first immunoprecipitated for c-Fos followed by second IP for β-catenin or other transcription factors or IgG. The difference is calculated between averages of three experiments. n.s., not significant; *, *P* < 0.05; **, *P* < 0.01, ***, *P* < 0.005.

ChIP assays were used to verify binding of the transcription factors to the promoter region of PTMA gene (the location of PCR primers is shown in Figure 5A). We found that β-catenin, TCF4, c-Fos, and CREB interacted with both AP1-binding sequence (AP1BS or 1500–1600-bp region) and TCF4-binding sequence (TCF4BS or 1000–1100-bp region) upstream of the transcription start site (Figure 6A). Significant decrease in the binding of these factors to both regions was observed following sorafenib treatment (10 μM), except for TCF4 in the TCF4BS region (Figure 6A and Supplementary Figure S3A). These results indicate that binding of β-catenin, TCF4, and c-Fos to AP1BS is inhibited by sorafenib. Interestingly, TCF4 interacted better with AP1BS than TCF4BS (over 10 fold). However, TCF4 binding at TCF4BS was increased, and binding at the AP1BS was inhibited by sorafenib. These results suggest that TCF4 binding to AP1BS may occur through formation of a complex with AP-1 and/or β-catenin. c-Myc binding was used as a control that could be detected at the TCF4BS region but not at AP1BS [6]. These binding activities were not detected in the exon-5 region where no such binding sequences exist. Binding of the β-catenin/TCF4/c-Fos complex to the PTMA promoter and inhibition by sorafenib treatment (10 μM) was also investigated using re-ChIP assay. IP was performed from nuclear extracts first with the β-catenin or c-Fos antibody, followed by treatment with the indicated second antibodies. The results revealed binding of β-catenin/TCF4 or β-catenin/c-Fos complexes to both AP1BS and TCF4BS in Mahlavu and J7 cells, and these effects were significantly reduced by sorafenib (*P* < 0.05; Figure 6B and Supplementary Figure S3B). We noted that β-catenin/TCF4 binding at AP1BS was 3.5 fold higher than binding at TCF4BS. As seen in ChIP assays, the binding results observed in re-ChIP assays were not detected in the negative control immunoprecipitated with either IgG or PCR at exon 5. In addition, β-catenin did not interact with CREB in CREB-binding element of the PTMA gene, indicating that CREB was not involved in this regulation. In contrast, using c-Fos as first antibody to conduct IP followed by β-catenin as second antibody also resulted in strong binding to the AP1BS, and less binding to the TCF4BS. Both β-catenin/c-Fos binding were inhibited by sorafenib in Mahlavu and J7 cells (Figure 6C and Supplementary Figure S3C). These results suggest that down-regulation of PTMA transcription by sorafenib may depend on β-catenin/TCF4/AP1 complex binding to the enhancer AP1BS as well as TCF4BS.

### Inhibition of AP-1 down-regulates PTMA protein and mRNA levels and potentiates sorafenib-induced apoptosis

**Figure 7:**
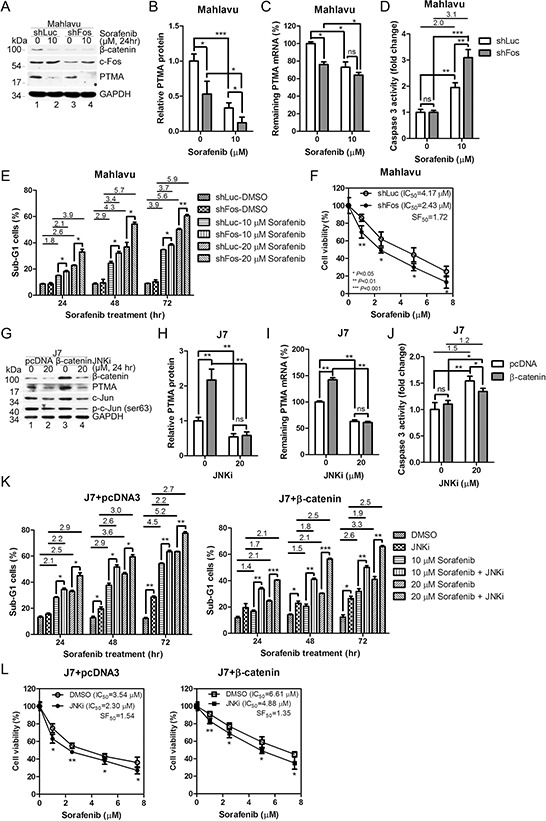
β-catenin and c-Fos are involved in the regulation of PTMA gene and HCC cell sensitivity by sorafenib **A.** Reduction of PTMA and β-catenin protein levels by c-Fos silencing. Noted that both PTMA and β-catenin, but not c-Fos, protein levels were inhibited by sorafenib. **B.** Quantification of PTMA protein reduction by c-Fos silencing of (A). **C.** Reduction of PTMA mRNA level by c-Fos silencing. **D.** Increased activation of caspase-3 by sorafenib in Mahlavu cells following c-Fos silencing. **E.** Enhancement of sorafenib-induced sub-G1 cell accumulation following c-Fos silencing. Fold change between treatments is indicated. **F.** Sensitization of Mahlavu cells to sorafenib following c-Fos silencing. Cell sensitivity was assessed using the MTT assay. SF_50_ was calculated by dividing the IC_50_ of control shLuc cells by that of shFos cells. **G.** Accumulation of PTMA protein level by β-catenin overexpression and its reduction by JNK inhibitor in J7 cells. Noted that both PTMA and β-catenin protein levels were inhibited by JNK inhibitor. **H.** Quantification of the reduction of β-catenin-induced PTMA protein level JNK inhibitor of (G). **I.** Reduction of β-catenin-induced PTMA mRNA level by JNK inhibition/c-Fos silencing in J7 cells. **J.** Reduced caspase-3 activity by 20 μM JNKi in J7 cells following β-catenin overexpression. **K.** Reduced sorafenib-induced sub-G1 cell accumulation following β-catenin overexpression (right panel) or pcDNA3 control (left panel) and reversal by JNK inhibition/c-Fos silencing. **L.** Protection of Mahlavu cells against sorafenib following β-catenin overexpression and reversal by JNK inhibition/c-Fos silencing. Cell viability was assessed using the MTT assay. Differences are calculated as averages of three experiments. Symbols are the same as for Figure 2.

Our previous study showed that the JNK pathway is involved in PTMA expression [6]. To verify if the effects of β-catenin on PTMA transcription involves JNK/AP1, we performed experiments to inhibit JNK/AP1. First, we examined whether c-Fos regulates PTMA expression. PTMA protein and mRNA levels decreased following c-Fos silencing in Mahlavu cells compared to shLuc-expressing cells (Figure 7A– 7C). β-catenin protein levels also decreased after c-Fos silencing (Figure 7A). While β-catenin level was dramatically reduced by sorafenib, the level of c-Fos protein remained unchanged (Figure 7A, compare lanes 1 and 2; lanes 3 and 4). Caspase-3 activation was enhanced by sorafenib treatment in cells in which c-Fos was silenced compared to shLuc expressing cells (Figure 7D). Furthermore, induction of sub-G1 cells and inhibition of cell viability by sorafenib were potentiated by c-Fos silencing in Mahlavu cells (Figure 7E and 7F). These results indicate that silencing of c-Fos reduces β-catenin and PTMA expression levels and sensitizes cells to sorafenib.

To assess the importance of JNK/AP1 in β-catenin-mediated regulation of PTMA expression, we inhibited AP1 activity in J7 cells using JNK inhibitor (JNKi). β-Catenin and PTMA protein and mRNA levels were reduced by JNKi in cells expressing the pcDNA3 vector or β-catenin (Figure 7G–7I). Notably, increase of PTMA protein and mRNA levels was prevented following ectopic expression of β-catenin and JNKi treatment. While caspase-3 activity increased following JNKi treatment in J7 cells, ectopic expression of β-catenin moderately reduced caspase-3 activity (Figure 7J). Sub-G1 cells increased in a dose-dependent manner in sorafenib-treated cells. JNKi treatment further enhanced the levels of sub-G1 cells in the J7 cell line (Figure 7K, left panel). Ectopic expression of β-catenin reversed the level of sub-G1 cells compared to control vector (Figure 7K, right panel). While cell viability decreased in a dose-dependent manner following sorafenib treatment in J7 cells, JNKi treatment sensitized the cells to the cytotoxic effects of sorfenib in cells overexpressing either the vector or β-catenin (Figure 7L, left panel, SF_50_ = 1.54, and right panel, SF_50_ = 1.35, respectively). IC_50_ values of 3.54 μM and 6.61 μM in control and β-catenin-overexpressing cells were observed, respectively, with resistance factor of 1.87, indicating the protective role of β-catenin in this context. Resistance factor of 2.12 (IC_50_ = 4.88 divided by IC_50_ = 2.30) in β-catenin-overexpressing cells was observed in the presence of JNKi. These effects were unlikely to represent off-target activities since the specificity of JNK inhibitor was tested in Mahlavu cells (Supplementary Figure S4). These results suggest that PTMA expression is stimulated by JNK-dependent β-catenin expression, and that the expression level of these genes is critical to determine cell sensitivity to sorafenib.

### Sorafenib potentiates the degradation of β-catenin mRNA

**Figure 8:**
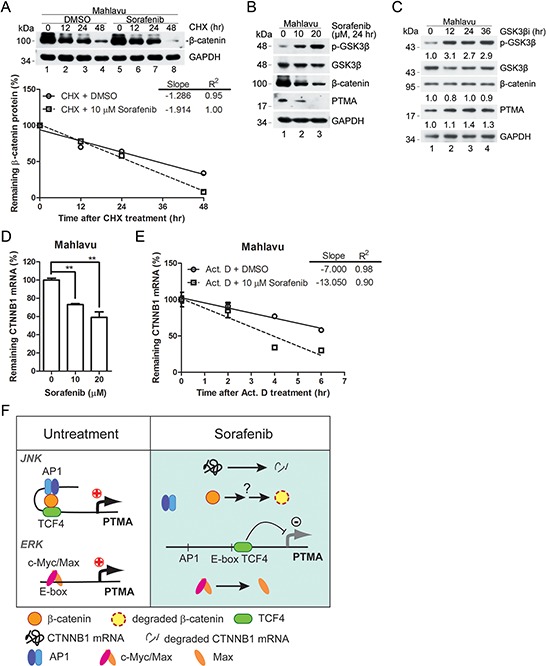
Effects of GSK3β signal pathway on β-catenin and PTMA expression **A.** Degradation rate of β-catenin protein in sorafenib-treated Mahlavu cells. Cells were pre-incubated with cycloheximide (CHX), an inhibitor of protein synthesis, followed by the indicated incubation. Linear regressions of the kinetic pattern of PTMA protein level are shown at bottom. **B.** Decreased GSK3β signaling in sorafenib-treated Mahlavu cells. GSK3β inactivation is shown based on the phosphorylated form of the kinase by sorafenib in a dose-dependent manner. Simultaneous decrease of PTMA protein level was observed in the same treatment cells. **C.** Lack of reduction of β-catenin and PTMA protein level following treatment with GSK3β inhibitor (GSK3βi) for the indicated time. Cells were treated with 50 nM of GSK3β inhibitor for the time indicated. Fold change, relative to untreated control, is indicated. **D.** Reduction of CTNNB1 mRNA level by sorafenib in Mahlavu cells. **E.** Increased degradation rate of the CTNNB1 mRNA in sorafenib-treated Mahlavu cells. Cells were pre-incubated with actinomycin D (Act. D), an inhibitor of RNA synthesis, followed by the indicated incubation. Results are shown as linear regression of the kinetic pattern of CTNNB1 mRNA level. **F.** Working model for the involvement of the β-catenin/JNK/PTMA and ERK/c-Myc/Max/PTMA axes in HCC sensitivity to sorafenib. In the absence of sorafenib (left panel, untreatment), β-catenin acts in concert with TCF4 and the AP-1/JNK pathway to stimulate PTMA transcription. In the presence of sorafenib (right panel), β-catenin was degraded and the components AP-1/TCF4 were dissociated, leading to downregulation of PTMA transcription. The ERK/c-Myc/Max axis in regulation of PTMA previously identified [6] is also included.

The protein level of β-catenin following treatment with cycloheximide (CHX), an inhibitor of protein synthesis, for 12 or 24 hrs did not vary in the absence or presence of sorafenib (Figure 8A). However, different protein levels were observed after 48 hrs of CHX treatment (Figure 8A), indicating that the degradation rate of β-catenin was slightly enhanced by sorafenib. Previous studies have shown that dysregulation of the Wnt pathway which involves β-catenin and p-GSK3β is critical for β-catenin degradation in HCC [15]. To further assess the regulation of β-catenin expression by sorafenib, we examined the possible upstream kinase signal pathway that may regulate β-catenin expression. While the protein levels of β-catenin and PTMA were down-regulated in a dose-dependent manner by sorafenib (24 hrs) in Mahlavu cells, the phosphorylated form of GSK3β(p-GSK3β) increased (Figure 8B).

We also found a slight increase of PTMA protein level in cells treated with the GSK3β inhibitor, whereas β-catenin protein levels remained unchanged (Figure 8C). These results suggest that the decrease of PTMA in sorafenib-treated Mahlavu cells is unlikely to be due to enhanced degradation of β-catenin via GSK3β activity. On the other hand, we found decreased β-catenin mRNA levels and enhanced mRNA degradation rate following sorafenib treatment as revealed by the reduced mRNA levels in cells treated with the transcription inhibitor actinomycin D (Act. D) (Figure 8D and 8E). While the total levels of β-catenin mRNA and protein were down-regulated by JNK inhibitor, the nuclear translocation of β-catenin protein and the degradation rates of β-catenin mRNA and protein were not affected (Supplementary Figure S5). These results demonstrate that down-regulation of β-catenin expression by sorafenib is controlled by enhancement of mRNA degradation, in addition to inhibition of transactivation of the gene.

### High levels of β-catenin and PTMA in clinical tumors

Whether our findings in cell lines can be extrapolated to clinical samples and cancer patients remains to be examined. However, we observed that RNA levels of CTNNB1, PTMA, and c-Myc correlate with poor survival in HCC patients (Supplementary Figure S6A–S6C). In addition, the expression level of CTNNB1 and PTMA was higher in HCC than in control healthy liver tissues (Supplementary Figure S6D–S6E). As for CTNNB1 and PTMA, the expression level of c-Myc was also higher in advanced HCC compared to early tumors (Supplementary Figure S6F). Apparently, the observation that RNA levels of CTNNB1 and PTMA were higher in HCC than in control healthy liver tissues could not fully explain cancer sensitivity to the drug. In addition, correlation between β-catenin and PTMA mRNA levels was poor in these clinical tumors (Supplementary Figure S6G, S6H). A study of the nuclear expression of β-catenin protein and/or its activity is required to elucidate the possible role of this protein in regulation of PTMA expression and sorafenib sensitivity in clinical tumors. Although only a few HCC cell lines were tested in this study, these cell lines (except Huh7) displayed good correlation between β-catenin activity and PTMA protein levels. This observation suggests that their expression level may serve a potential marker of sorafenib sensitivity.

## DISCUSSION

In this study, we found that down-regulation of PTMA in response to sorafenib occurs through inhibition of the β-catenin pathway and that this process contributes to the cell killing effects of sorafenib in HCC cells. Induction of caspase-9 and caspase-3 and the cell killing effects of sorafenib are associated with PTMA levels in HCC cells. Furthermore, silencing of β-catenin results in down-regulation of PTMA expression and cell sensitization to the drug, effects which can be rescued by PTMA overexpression. These observations support the conclusion that the β-catenin/PTMA pathway plays an important role in modulating HCC sensitivity to sorafenib. We further demonstrated that the regulation of PTMA expression by β-catenin occurs at the transcription level, specifically in processes involving gene transactivation and maintenance of mRNA stability. As evidenced by the gain-of-function and loss-of-function experiments described here, we identified that the PTMA gene is activated by β-catenin in cooperation with AP-1 targeting to the AP-1 enhancer of the PTMA gene segment where the mediator TCF4 [15] is also involved. In the presence of sorafenib, β-catenin is likely to be down-regulated at the transcription level, and the β-catenin/AP1/TCF4 complex may fail to form, leading to inhibition of PTMA gene transactivation (Figure 8F). During this process, TCF4 is also involved in β-catenin/AP-1 complex binding to AP1BS for PTMA gene activation. Interestingly, the binding of TCF4 to AP1BS in the PTMA gene is stronger than the binding to TCF4BS. In the presence of sorafenib, the binding of TCF4 to AP1BS is inhibited whereas binding to TCF4BS is induced (Figure 6A). This is probably due to down-regulation of β-catenin/AP-1 by the drug since β-catenin/AP-1 complex binding to AP1BS is sensitive to the drug (Figure 6B). Thus, activation of PTMA gene involves the β-catenin/AP-1/TCF4 complex and is negatively regulated by sorafenib in HCC cells. Our findings provide evidence linking the surviving/oncogenic protein β-catenin to the antiapoptotic factor PTMA. Inhibition of this axis sensitizes HCC cells to sorafenib, suggesting that this strategy may help control the growth of this cancer.

We also found that sorafenib down-regulates PTMA expression in HCC cells, a process which depends on nuclear accumulation of β-catenin, a hallmark of Wnt pathway activation. While phosphorylation of GSK3βincreases following sorafenib treatment, β-catenin and PTMA are down-regulated in sorafenib-treated HCC cells (Figure 8). The canonical Wnt/β-catenin signaling pathway appears to be a prominent target of sorafenib in HCC. Indeed, 40–70% of HCCs harbor nuclear accumulation of the β-catenin protein [38–40]. As seen for other tumors, Wnt/β-catenin signaling is a critical contributor to HCC pathogenesis, and proteins identified in this pathway are potential candidates for pharmacological interventions [15]. However, our results did not elucidate the effects of β-catenin mutations which may affect cell functions such as oncogenesis and treatment efficacy. Activating mutations in the β-catenin gene (CTNNB1) occur in 8–30% of tumors, while loss-of-function/mutations in APC and Axin genes occur in 1–3% and 8–15%, respectively, and are mutually exclusive to CTNNB1 mutations [10, 38, 41–45]. While some observations suggest that CTNNB1 mutations could be a late event during hepatocarcinogenesis, β-catenin accumulation was detected in the early stage during the development of HCC, suggesting that other mechanisms could contribute to β-catenin stabilization [43, 46]. The cell lines used in the present study were derived from advanced HCC cases and they express various levels of β-catenin. Although silencing CTNNB1 (which led to reduction of PTMA levels) sensitized HCC cells to sorafenib, we still do not know whether CTNNB1 mutations occurred in these HCC cell lines.

A recent review summarized the potential Wnt-component targets for therapeutic intervention on tumor development and growth [15]. While β-catenin activation may cooperate with non-canonical pathways such as insulin/IGF-1/IRS-1/MAPK during hepatocarcinogenesis in mice [47], our study on sorafenib-induced apoptosis in HCC cell lines reveals the involvement of β-catenin and MAPK/AP-1. Inhibition of AP-1 by shRNA or chemical inhibitor down-regulates β-catenin protein levels, suggesting that β-catenin acts downstream of AP-1. These two pathways may be muturally regulated and thus be impaired by downregulation of either one. Accordingly, β-catenin activation may cooperate with MAPK/AP-1 to potentiate PTMA transcription. In this context, the mechanisms underlying the development of chemoresistance may partly overlap with that of β-catenin-associated oncogenesis. Additionally, we found that oncogenic c-Myc activates PTMA and represents a potent target of sorafenib in HCC cells [6]. These results support the notion that activation of oncogenes and/or inactivation of tumor suppressor genes during tumor development reduce treatment sensitivity, and are potential therapeutic targets to reduce chemoresistance [48].

We present new findings linking β-catenin activity with expression of the PTMA protein. We showed previously that PTMA modulates the sensitivity of HCC cells to the kinase inhibitor sorafenib [6]. Sorafenib was shown to downregulate β-catenin and β-catenin activity through the JNK pathway, a process which was associated with suppressed PTMA activity. We also found that CTNNB1 mRNA level is downregulated by sorafenib through inhibition of the AP-1/JNK pathway, possibly via transactivation inhibition. However, the mechanism by which β-catenin is altered by sorafenib remains unclear. These cellular processes enhance apoptosis and partly overcome drug resistance. A simplified model is presented and includes the effects of the β-catenin/AP-1/TCF4 axis and the c-Myc/Max axis in the regulation of PTMA transactivation (Figure 8F). Nevertheless, how these molecules interact and contribute to PTMA expression still remains to be elucidated. As for the down-regulation of PTMA, we also found that sorafenib down-regulates the anti-apoptotic proteins survivin and Mcl-1 in HCC cells. We have previously demonstrated the protective role of HURP (which acts upstream of survivin) and Mcl-1 against sorafenib in HCC and endometrial carcinoma cells, respectively [3, 49]. The possible involvement of these proteins in modulating sorafenib response in HCC cells and the link between such proteins and β-catenin need to be examined in further studies.

In conclusion, our observation that PTMA plays a critical role in HCC progression and drug resistance is an important finding that may lead to the development of novel cancer therapies. Compounds such as sorafenib that potently interfere with signaling pathways and effectors (e.g., PTMA) that are required for HCC progression may be used to treat selected human patients and improve current therapies against HCC.

## MATERIALS AND METHODS

### Cell cultures and reagents

Primary Chang liver cells and hepatocellular carcinoma cells (Huh7, J7, SK-Hep1, and Mahlavu; kindly provided by Prof. K.-H. Lin, Chang Gung University) were maintained in Dulbecco's modified Eagle's medium (DMEM; Gibco, Gaithersburg, MD, USA) supplemented with 10% (v/v) fetal bovine serum (FBS), penicillin (100 U/ml; Gibco), and streptomycin (100 mg/ml; Gibco). All cells were incubated at 37°C in a humidified atmosphere of 5% CO_2_ (v/v) in air. The reagents used included antibodies against Bad, pBad, Bid, β-catenin, pc-Jun, CREB, pGSK3β, GSK3β (Cell Signaling, Danvers, MA, USA), PUMA, PTMA, survivin, Mcl-1, Bcl-X_L_, Bcl-2, Bax, Bim, GAPDH, lamin A/C, VDAC, c-Myc, FLIP, caspase-8, and c-Jun/AP1 (Santa Cruz Biotechnology, Santa Cruz, CA, USA). Kinase inhibitors of JNK (SP600125) (Santa Cruz Biotechnology) and GSK3β (GSK3β inhibitor II; Cell Signaling) were also used. Sorafenib (Bayer Health Care AG, Berlin, Germany) was kindly provided by Dr. T.-C. Chang (Chang Gung Memorial Hospital, Taoyuan, Taiwan). The other chemicals were purchased from Sigma-Aldrich.

### Plasmids and cell transfection

The pcDNA3 plasmid was used as a negative control. pcDNA3-PTMA and a 2,500-bp functional promoter segment of the PTMA gene isolated from Mahlavu cell genomic DNA and 5′-truncated promoter segments of PTMA, inserted in the pGL3-basic reporter vector (Promega), were constructed as previously described [6]. The pcDNA3-β-catenin expression plasmid was kindly provided by Dr. J.J.-C. Lin (Department of Biology, University of Iowa, Iowa City, IA, USA) [50]. Site-specific mutations of the PTMA promoter were constructed by PCR-directed mutagenesis using synthetic, mismatched oligonucleotides, resulting in pGL3-PTMA-AP1(mt) [51], pGL3-PTMA-TCF4(mt) [52], and pGL3-PTMA-CREB(mt) [53]. Plasmid construction and preparation was performed according to standard protocols [54]. Cells were transfected with plasmids using Lipofectamine (Invitrogen, Carlsbad, CA, USA) according to the instructions provided by the supplier. Transfected cells were incubated 48 hrs for overexpression of the plasmids.

### Quantitative real-time reverse transcription-PCR (qRT-PCR)

qRT-PCR, or in short qPCR, was performed on total RNA extracted with Trizol (Invitrogen) and 200 nM of primers as described [55]. The primers used were as follows: PTMA, forward, 5′-CGAAATCACCACCAAGGACT-3′; reverse, 5′-GTCGGTCTTCTGCTTCTTGG-3′; and CTNNB1, forward, 5′-GGCGCCATTTTAAGCCTCTC-3′; reverse, 5′-TGGCCATGTCCAACTCCATC-3′; and GAPDH, forward, 5′-TCCTGCACCACCAACTGCTT-3′; reverse, 5′-GAGGGGGCCATCCACGTCTT-3′. All unknown samples and controls were done in triplicate. Relative quantification was calculated using the ΔΔCt method and normalized against GAPDH as before [56]. Namely, the ΔCt for each candidate was calculated as ΔCt (candidate) = [Ct (candidate) – Ct (GAPDH)]. Relative abundance of the candidate gene X was shown as 2^ΔCt(X) – ΔCt(GAPDH)^.

### Western blot analysis

Whole cell protein extracts were prepared for immunoblotting as before [57]. Protein concentration was determined using the Bradford assay and the BioRad dye reagent (BioRad, Hercules, CA, USA). Proteins (50 μg) from each sample were separated by sodium dodecyl sulfate-polyacrylamide gel electrophoresis (SDS-PAGE), transferred onto PVDF membranes, prior to incubation with antibodies according to the instructions of the manufacturer. Protein signals were revealed using enhanced chemiluminescence according to the specifications of the supplier (Pierce, Rockford, IL, USA). Intensity of the protein bands was determined using a scanning densitometer (Personal Densitometer SI: Amersham Biosciences, Sunnyvale, CA, USA).

### Silencing of selected genes using short-hairpin RNA

pLKO.1 plasmids expressing shRNA were purchased from the National RNAi Core Facility. Luciferase shRNA (TRCN0000072244) was used as a negative control. Transient transfection was done by adding 2 μg/well (unless indicated otherwise) of shRNA plasmids along with 5 μl/well of Lipofectamine (Invitrogen) into cell suspensions kept in six-well plates (1.5 (10^4^ cells/well) as described before [56]. The plasmids used included PTMA (TRCN0000135421), CTNNB1/β-catenin (TRCN0000314991), and c-Fos (TRCN0000016004). Stable clones expressing shRNA plasmids via lentivirus as vector were established in HCC cells.

### Fractionation of nucleus and cytosol

Cells were washed once with PBS, prior to lysis in extraction buffer (10 mM KCl, 20 mM HEPES, pH 7.9, 1.5 mM MgCl_2_, 0.2 mM EDTA, 20% glycerol, 0.5 mM DTT). Following incubation on ice for 15 min, cell lysates were mixed by pipeting several times and centrifuged 1 min at 14,000 rpm to pellet nuclei. The supernatant was centrifuged under the same conditions. The final supernatant was partitioned in tubes and centrifuged 5 min at 14,000 rpm. The supernatant containing cytosolic proteins was collected. The nuclear pellet was washed once with extraction buffer, prior to lysis in nuclear extraction buffer (500 mM KCl, 20 mM HEPES, pH 7.9, 1.5 mM MgCl_2_, 0.2 mM EDTA, 10% glycerol, 0.5 mM DTT, and protease inhibitor cocktail; BD Biosciences, San Jose, CA, USA) and centrifuged 5 min as above. The entire purification process was performed at 4°C.

### Analysis of cell viability and apoptosis

Cells were treated with sorafenib in culture medium for three days unless indicated otherwise. Cell viability was determined using the 3-(4,5-dimethylthiazol-2-yl)-2,5-diphenyltetrazolium bromide (MTT) colorimetric assay [58]. Percentage of viable cells was calculated as the ratio of OD_570 nm_ values for treated cells divided by the OD_570 nm_ values for control cells. To evaluate apoptosis, sub-G1 cells were measured as before [59]. Stained nuclei were analyzed using the BD FACScan Flow Cytometer (Becton & Dickinson, San Jose, CA, USA) with 10,000 events/determination. The LYSYS II software was used to assess cell cycle distribution. Unless indicated otherwise, samples were prepared in quadruplicate and three independent experiments were performed.

### Caspase activity assay

Caspase-3 and caspase-9 activity was monitored using a commercial colorimetric assay kit (BioVision, Milpitas, CA). Cells were treated with sorafenib (10 and 20 μM), JNKi (20 μM), or DMSO for 24 hrs. Cells were resuspended in 50 μl of cold cell lysis buffer and incubated on ice for 10 min. Following centrifugation for 1 min at 12,000 rpm, supernatants corresponding to cytosolic extracts were transferred to new tubes and incubated on ice for immediate assay or aliquoted and stored at −80°C for future use. Cytoplasmic proteins (100 μg) and 5 μl of substrate were added to 50 μl of 2× reaction buffer, prior to incubation at 37°C for 2 hrs. Color was read at OD_405 nm_ in a micro-plate reader.

### Luciferase promoter analysis

HCC cells were transfected with 2 μg of pGL3-PTMA reporter plasmid. Two μg of the Renilla luciferase plasmid pGL4.74 [hRluc/TK] (Promega; kindly provided by Dr. J. Horng, Chang Gung University) was included with each reporter plasmid for normalization. HCC cells transfected with the plasmids were incubated 48 hrs. Transcriptional activity was monitored using a dual luciferase reporter assay (Promega) and a luminescence reader (LMaxII384, Molecular Devices, Sunnyvale, CA, USA) according to the instructions provided by the manufacturers. For each experiment, luciferase activity was normalized to Renilla luciferase.

### Chromatin immunoprecipitation (ChIP) assay

Formaldehyde cross-linking and ChIP assays of tissue culture cells were performed based on a modified protocol [60] as described before [61] using a commercial kit (Upstate Biotechnology, Lake Placid, NY, USA). Chromatin was sonicated on ice to obtain DNA fragments of 0.3–1.5 kbp and averaging 600 bp. Twenty percent of total supernatant was used as a total-input control. Following removal of bound proteins, immuno-precipitated DNA was subjected to qPCR or regular PCR (35 cycles). The products amplified by regular PCR were separated on a 1.5%-agarose gel and visualized using ethidium bromide staining. AP-1 (activator protein-1) and c-Myc-binding sites on the endogenous PTMA promoter on chromosome 2 (NC_000002) were predicted using TFSearch (http://www.cbrc.jp/research/db/TFSEARCH.html; Tokyo, Japan). Primers were designed using Primer 3.0 (Life Technologies, Carlsbad, CA, USA). TCF4 and CREB-binding sites were described previously [53, 62, 63]. The primers used included: primer pair for c-Myc and TCF4-binding site in the promoter region, forward, 5′-TTCTGGTCCTTTTTCCCACA-3′ and reverse, 5′-TTTAGAGAACCATGCGGAGC-3′, yielded a 200-bp product; primer pair for AP-1 and CREB-binding site, forward, 5′-AGACTGCGTGCTAAGCTC-3′ and reverse, 5′-AGCTGGGAATGGGGAAAAC-3′, yielded a 178-bp product; primer pair 3 for the exon-5 region, forward, 5′-GATGACACGCGCTCTCCAC-3′ and reverse, 5′-TCCGAAGGCTGGTTTGGTCA-3′, produced a 214-bp product. Re-ChIP was performed with the same procedure using double immunoprecipitation with two antibodies before PCR. ChIP-qPCR was performed using a qPCR kit and the same primer pairs. The ΔCt for each candidate was calculated as ΔCt (normalized ChIP) = [Ct (ChIP) – Ct (Input)]. The % input was shown as 2^[−ΔCt(normalized ChIP)]^.

### Statistical analysis

Data were reported as mean values ± Standard deviation (SD). Three independent experiments were performed unless indicated otherwise. Statistical significance (*p* value) was calculated with a two-tailed Student's *t* test for single comparison. The symbols *, **, and *** denote *p* < 0.05, *p* < 0.01 and *p* < 0.001, respectively.

## SUPPLEMENTARY FIGURES



## Abbreviations

C/EBPβ: CCAAT/enhancer-binding protein beta
ChIP: chromatin immunoprecipitation
CREB: c-AMP responsive element binding protein
DMSO: dimethyl sulfoxide
ERK: extracellular regulated protein kinase
FBS: fetal bovine serum
GAPDH: glyceraldehyde 3-phosphate dehydrogenase
GFP: green fluorescent protein
HCC: hepatocellular carcinoma
HURP: hepatoma upregulated protein
JNK: c-Jun N-terminal kinases
Luc: luciferase
MAPK: mitogen-activated protein kinase
MTT: 3-(4,5-dimethylthiazol-2-yl)-2,5-diphenyltetrazolium bromide
PARP: poly (ADP-ribose) polymerase
PBS: phosphate-buffered saline
PCR: polymerase chain reaction
PTMA: prothymosin alpha
PVDF: polyvinylidene fluoride
qRT-PCR: quantitative real-time reverse transcription-PCR
SDS-PAGE: sodium dodecyl sulfate-polyacrylamide gel electrophoresis
shRNA: short hairpin RNA
VDAC: voltage-dependent anion channels.

## References

[1] Parkin DM, Bray F, Ferlay J, Pisani P (2005). Global cancer statistics, 2002. CA: a cancer journal for clinicians.

[2] Jiang X, Kim H-E, Shu H, Zhao Y, Zhang H, Kofron J, Donnelly J, Burns D, Ng S-c, Rosenberg S, Wang X (2003). Distinctive roles of PHAP proteins and prothymosin-alpha in a death regulatory pathway. Science.

[3] Kuo TC, Lu HP, Chao CC (2011). The tyrosine kinase inhibitor sorafenib sensitizes hepatocellular carcinoma cells to taxol by suppressing the HURP protein. Biochemical pharmacology.

[4] Tsou AP, Yang CW, Huang CY, Yu RC, Lee YC, Changx CW, Chen BR, Chung YF, Fann MJ, Chi CW, Chiu JH, Chou CK (2003). Identification of a novel cell cycle regulated gene, HURP, overexpressed in human hepatocellular carcinoma. Oncogene.

[5] Wu CG, Habib NA, Mitry RR, Reitsma PH, van Deventer SJ, Chamuleau RA (1997). Overexpression of hepatic prothymosin alpha, a novel marker for human hepatocellular carcinoma. British journal of cancer.

[6] Lin YT, Lu HP, Chao CC (2015). Oncogenic c-Myc and prothymosin-alpha protect hepatocellular carcinoma cells against sorafenib-induced apoptosis. Biochemical pharmacology.

[7] Polakis P (1999). The oncogenic activation of beta-catenin. Current opinion in genetics & development.

[8] Farazi PA, DePinho RA (2006). Hepatocellular carcinoma pathogenesis: from genes to environment. Nat Rev Cancer.

[9] Shibata T, Aburatani H (2014). Exploration of liver cancer genomes. Nature reviews Gastroenterology & hepatology.

[10] de La Coste A, Romagnolo B, Billuart P, Renard CA, Buendia MA, Soubrane O, Fabre M, Chelly J, Beldjord C, Kahn A, Perret C (1998). Somatic mutations of the beta-catenin gene are frequent in mouse and human hepatocellular carcinomas. Proceedings of the National Academy of Sciences of the United States of America.

[11] Miyoshi Y, Iwao K, Nagasawa Y, Aihara T, Sasaki Y, Imaoka S, Murata M, Shimano T, Nakamura Y (1998). Activation of the beta-catenin gene in primary hepatocellular carcinomas by somatic alterations involving exon 3. Cancer research.

[12] Cieply B, Zeng G, Proverbs-Singh T, Geller DA, Monga SP (2009). Unique phenotype of hepatocellular cancers with exon-3 mutations in beta-catenin gene. Hepatology.

[13] Kan Z, Zheng H, Liu X, Li S, Barber TD, Gong Z, Gao H, Hao K, Willard MD, Xu J, Hauptschein R, Rejto PA, Fernandez J, Wang G, Zhang Q, Wang B (2013). Whole-genome sequencing identifies recurrent mutations in hepatocellular carcinoma. Genome research.

[14] Sun Y, Campisi J, Higano C, Beer TM, Porter P, Coleman I, True L, Nelson PS (2012). Treatment-induced damage to the tumor microenvironment promotes prostate cancer therapy resistance through WNT16B. Nature medicine.

[15] Pez F, Lopez A, Kim M, Wands JR, Caron de Fromentel C, Merle P (2013). Wnt signaling and hepatocarcinogenesis: molecular targets for the development of innovative anticancer drugs. Journal of hepatology.

[16] Wilhelm S, Chien DS (2002). BAY 43–9006: preclinical data. Current pharmaceutical design.

[17] Wilhelm SM, Carter C, Tang L, Wilkie D, McNabola A, Rong H, Chen C, Zhang X, Vincent P, McHugh M, Cao Y, Shujath J, Gawlak S, Eveleigh D, Rowley B, Liu L (2004). BAY 43–9006 exhibits broad spectrum oral antitumor activity and targets the RAF/MEK/ERK pathway and receptor tyrosine kinases involved in tumor progression and angiogenesis. Cancer research.

[18] Carlomagno F, Anaganti S, Guida T, Salvatore G, Troncone G, Wilhelm SM, Santoro M (2006). BAY 43–9006 inhibition of oncogenic RET mutants. Journal of the National Cancer Institute.

[19] Chang CJ, Yang JY, Xia W, Chen CT, Xie X, Chao CH, Woodward WA, Hsu JM, Hortobagyi GN, Hung MC (2011). EZH2 promotes expansion of breast tumor initiating cells through activation of RAF1-beta-catenin signaling. Cancer cell.

[20] Galuppo R, Maynard E, Shah M, Daily MF, Chen C, Spear BT, Gedaly R (2014). Synergistic inhibition of HCC and liver cancer stem cell proliferation by targeting RAS/RAF/MAPK and WNT/beta-catenin pathways. Anticancer research.

[21] Lachenmayer A, Alsinet C, Savic R, Cabellos L, Toffanin S, Hoshida Y, Villanueva A, Minguez B, Newell P, Tsai HW, Barretina J, Thung S, Ward SC, Bruix J, Mazzaferro V, Schwartz M (2012). Wnt-pathway activation in two molecular classes of hepatocellular carcinoma and experimental modulation by sorafenib. Clinical cancer research : an official journal of the American Association for Cancer Research.

[22] Muche S, Kirschnick M, Schwarz M, Braeuning A (2014). Synergistic effects of beta-catenin inhibitors and sorafenib in hepatoma cells. Anticancer research.

[23] Barbini L, Gonzalez R, Dominguez F, Vega F (2006). Apoptotic and proliferating hepatocytes differ in prothymosin alpha expression and cell localization. Molecular and cellular biochemistry.

[24] Mori M, Barnard GF, Staniunas RJ, Jessup JM, Steele GD, Chen LB (1993). Prothymosin-alpha mRNA expression correlates with that of c-myc in human colon cancer. Oncogene.

[25] Shibuta K, Mori M, Mimori K, Inoue H, Nakashima H, Baba K, Haraguchi M, Karimine N, Ueo H, Akiyoshi T (1996). Expression of prothymosin-alpha and c-myc mRNA in human gastric cancer. International journal of oncology.

[26] Sasaki H, Sato Y, Kondo S, Fukai I, Kiriyama M, Yamakawa Y, Fujii Y (2001). Expression of the prothymosin a mRNA correlated with that of N-myc in neuroblastoma. Cancer letters.

[27] Eilers M, Schirm S, Bishop JM (1991). The MYC protein activates transcription of the alpha-prothymosin gene. The EMBO journal.

[28] Gaubatz S, Meichle A, Eilers M (1994). An E-box element localized in the first intron mediates regulation of the prothymosin alpha gene by c-myc. Molecular and cellular biology.

[29] Mol PC, Wang R-H, Batey DW, Lee LA, Dang CV, Berger SL (1995). Do products of the myc proto-oncogene play a role in transcriptional regulation of the prothymosin alpha gene?. Molecular and cellular biology.

[30] Gaubatz S, Imhof A, Dosch R, Werner O, Mitchell P, Buettnerl R, Eilers M (1995). Transcriptional activation by Myc is under negative control by the transcription factor AP-2. The EMBO journal.

[31] Ponce DP, Yefi R, Cabello P, Maturana JL, Niechi I, Silva E, Galindo M, Antonelli M, Marcelain K, Armisen R, Tapia JC (2011). CK2 functionally interacts with AKT/PKB to promote the beta-catenin-dependent expression of survivin and enhance cell survival. Molecular and cellular biochemistry.

[32] Iqbal S, Zhang S, Driss A, Liu ZR, Kim HR, Wang Y, Ritenour C, Zhau HE, Kucuk O, Chung LW, Wu D (2012). PDGF upregulates Mcl-1 through activation of beta-catenin and HIF-1alpha-dependent signaling in human prostate cancer cells. PloS one.

[33] Shen L, Zhang X, Hu D, Feng T, Li H, Lu Y, Huang J (2013). Hepatitis B virus X (HBx) play an anti-apoptosis role in hepatic progenitor cells by activating Wnt/beta-catenin pathway. Molecular and cellular biochemistry.

[34] Augustin I, Goidts V, Bongers A, Kerr G, Vollert G, Radlwimmer B, Hartmann C, Herold-Mende C, Reifenberger G, von Deimling A, Boutros M (2012). The Wnt secretion protein Evi/Gpr177 promotes glioma tumourigenesis. EMBO molecular medicine.

[35] Ozawa M, Baribault H, Kemler R (1989). The cytoplasmic domain of the cell adhesion molecule uvomorulin associates with three independent proteins structurally related in different species. The EMBO journal.

[36] Nateri AS, Spencer-Dene B, Behrens A (2005). Interaction of phosphorylated c-Jun with TCF4 regulates intestinal cancer development. Nature.

[37] Toualbi K, Guller MC, Mauriz JL, Labalette C, Buendia MA, Mauviel A, Bernuau D (2007). Physical and functional cooperation between AP-1 and beta-catenin for the regulation of TCF-dependent genes. Oncogene.

[38] Lachenmayer A, Alsinet C, Savic R, Cabellos L, Toffanin S, Hoshida Y, Villanueva A, Minguez B, Newell P, Tsai HW, Barretina J, Thung S, Ward SC, Bruix J, Mazzaferro V, Schwartz M (2012). Wnt-pathway activation in two molecular classes of hepatocellular carcinoma and experimental modulation by sorafenib. Clin Cancer Res.

[39] Wong CM, Fan ST, Ng IO (2001). beta-Catenin mutation and overexpression in hepatocellular carcinoma: clinicopathologic and prognostic significance. Cancer.

[40] Huang H, Fujii H, Sankila A, Mahler-Araujo BM, Matsuda M, Cathomas G, Ohgaki H (1999). Beta-catenin mutations are frequent in human hepatocellular carcinomas associated with hepatitis C virus infection. The American journal of pathology.

[41] Laurent-Puig P, Legoix P, Bluteau O, Belghiti J, Franco D, Binot F, Monges G, Thomas G, Bioulac-Sage P, Zucman-Rossi J (2001). Genetic alterations associated with hepatocellular carcinomas define distinct pathways of hepatocarcinogenesis. Gastroenterology.

[42] Guichard C, Amaddeo G, Imbeaud S, Ladeiro Y, Pelletier L, Maad IB, Calderaro J, Bioulac-Sage P, Letexier M, Degos F, Clement B, Balabaud C, Chevet E, Laurent A, Couchy G, Letouze E (2012). Integrated analysis of somatic mutations and focal copy-number changes identifies key genes and pathways in hepatocellular carcinoma. Nature genetics.

[43] Park JY, Park WS, Nam SW, Kim SY, Lee SH, Yoo NJ, Lee JY, Park CK (2005). Mutations of beta-catenin and AXIN I genes are a late event in human hepatocellular carcinogenesis. Liver international : official journal of the International Association for the Study of the Liver.

[44] Taniguchi K, Roberts LR, Aderca IN, Dong X, Qian C, Murphy LM, Nagorney DM, Burgart LJ, Roche PC, Smith DI, Ross JA, Liu W (2002). Mutational spectrum of beta-catenin, AXIN1, and AXIN2 in hepatocellular carcinomas and hepatoblastomas. Oncogene.

[45] Amaddeo G, Guichard C, Imbeaud S, Zucman-Rossi J (2012). Next-generation sequencing identified new oncogenes and tumor suppressor genes in human hepatic tumors. Oncoimmunology.

[46] Suzuki T, Yano H, Nakashima Y, Nakashima O, Kojiro M (2002). Beta-catenin expression in hepatocellular carcinoma: a possible participation of beta-catenin in the dedifferentiation process. Journal of gastroenterology and hepatology.

[47] Longato L, de la Monte S, Kuzushita N, Horimoto M, Rogers AB, Slagle BL, Wands JR (2009). Overexpression of insulin receptor substrate-1 and hepatitis Bx genes causes premalignant alterations in the liver. Hepatology.

[48] Lowe SW, Lin AW (2000). Apoptosis in cancer. Carcinogenesis.

[49] Sun NK, Huang SL, Chang TC, Chao CC (2013). Sorafenib induces endometrial carcinoma apoptosis by inhibiting Elk-1-dependent Mcl-1 transcription and inducing Akt/GSK3beta-dependent protein degradation. Journal of cellular biochemistry.

[50] Wang Q, Lu TL, Adams E, Lin JL, Lin JJ (2013). Intercalated disc protein, mXinalpha, suppresses p120-catenin-induced branching phenotype via its interactions with p120-catenin and cortactin. Archives of biochemistry and biophysics.

[51] Risse G, Jooss K, Neuberg M, Bruller HJ, Muller R (1989). Asymmetrical recognition of the palindromic AP1 binding site (TRE) by Fos protein complexes. The EMBO journal.

[52] Lin SY, Xia W, Wang JC, Kwong KY, Spohn B, Wen Y, Pestell RG, Hung MC (2000). Beta-catenin, a novel prognostic marker for breast cancer: its roles in cyclin D1 expression and cancer progression. Proceedings of the National Academy of Sciences of the United States of America.

[53] Tinti C, Yang C, Seo H, Conti B, Kim C, Joh TH, Kim KS (1997). Structure/function relationship of the cAMP response element in tyrosine hydroxylase gene transcription. The Journal of biological chemistry.

[54] Sambrook J, Russell D.W (2001). Molecular cloning: a laboratory manual.

[55] Sun CL, Chao CC (2005). Cross-resistance to death ligand-induced apoptosis in cisplatin-selected HeLa cells associated with overexpression of DDB2 and subsequent induction of cFLIP. Molecular pharmacology.

[56] Wu ZZ, Lu HP, Chao CC (2010). Identification and functional analysis of genes which confer resistance to cisplatin in tumor cells. Biochemical pharmacology.

[57] Kamarajan P, Sun NK, Sun CL, Chao CC (2001). Apaf-1 overexpression partially overcomes apoptotic resistance in a cisplatin-selected HeLa cell line. FEBS letters.

[58] Chao CC, Huang SL, Huang HM, Lin-Chao S (1991). Cross-resistance to UV radiation of a cisplatin-resistant human cell line: overexpression of cellular factors that recognize UV-modified, DNA. Molecular and cellular biology.

[59] Tsai SY, Sun NK, Lu HP, Cheng ML, Chao CC (2007). Involvement of reactive oxygen species in multidrug resistance of a vincristine-selected lymphoblastoma. Cancer science.

[60] Shang Y, Hu X, DiRenzo J, Lazar MA, Brown M (2000). Cofactor dynamics and sufficiency in estrogen receptor-regulated transcription. Cell.

[61] Wu ZZ, Chow KP, Kuo TC, Chang YS, Chao CC (2011). Latent membrane protein 1 of Epstein-Barr virus sensitizes cancer cells to cisplatin by enhancing NF-kappaB p50 homodimer formation and downregulating NAPA expression. Biochemical pharmacology.

[62] Hallikas O, Palin K, Sinjushina N, Rautiainen R, Partanen J, Ukkonen E, Taipale J (2006). Genome-wide prediction of mammalian enhancers based on analysis of transcription-factor binding affinity. Cell.

[63] Bottomly D, Kyler SL, McWeeney SK, Yochum GS (2010). Identification of {beta}-catenin binding regions in colon cancer cells using ChIP-Seq. Nucleic acids research.

