# Supplementary material for: Identification of the β-catenin/JNK/prothymosin-alpha axis as a novel target of sorafenib in hepatocellular carcinoma cells
Source: Oncotarget. 2015 Oct 22;6(36):38999–9017. doi: 10.18632/oncotarget.5738 (PMC4770752; doi:10.18632/oncotarget.5738)
Supplement: Supplementary file 1 [file oncotarget-06-38999-s001.pdf]

SUPPLEMENTARY FIGURES

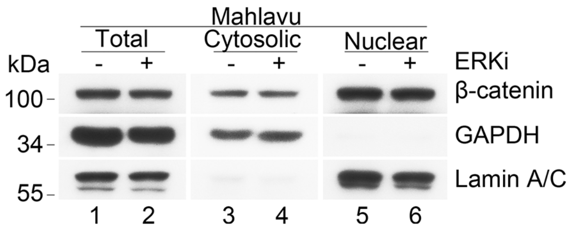

Supplementary Figure S1: The ERK pathway doe not regulate β-catenin transactivation activity.

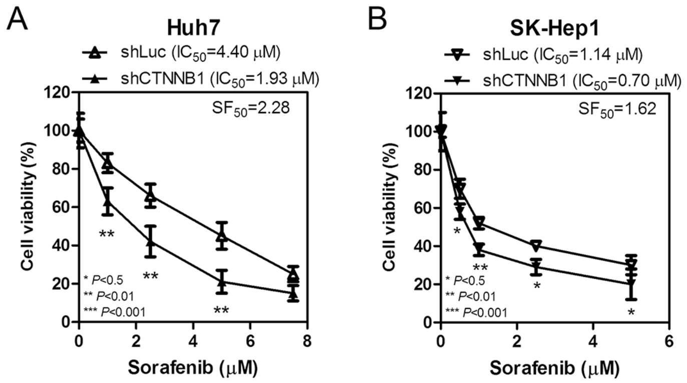

Supplementary Figure S2: Sensitization of Huh7 and SK-Hep1 cells to sorafenib following β-catenin silencing. The same experimental strategy shown in Figure 2D for β-catenin silencing was used in Huh7 **A.** and SK-Hep1 cells **B.** Results shown are expressed as means ± SD for three independent experiments. Symbols are the same as in Figure 2D.

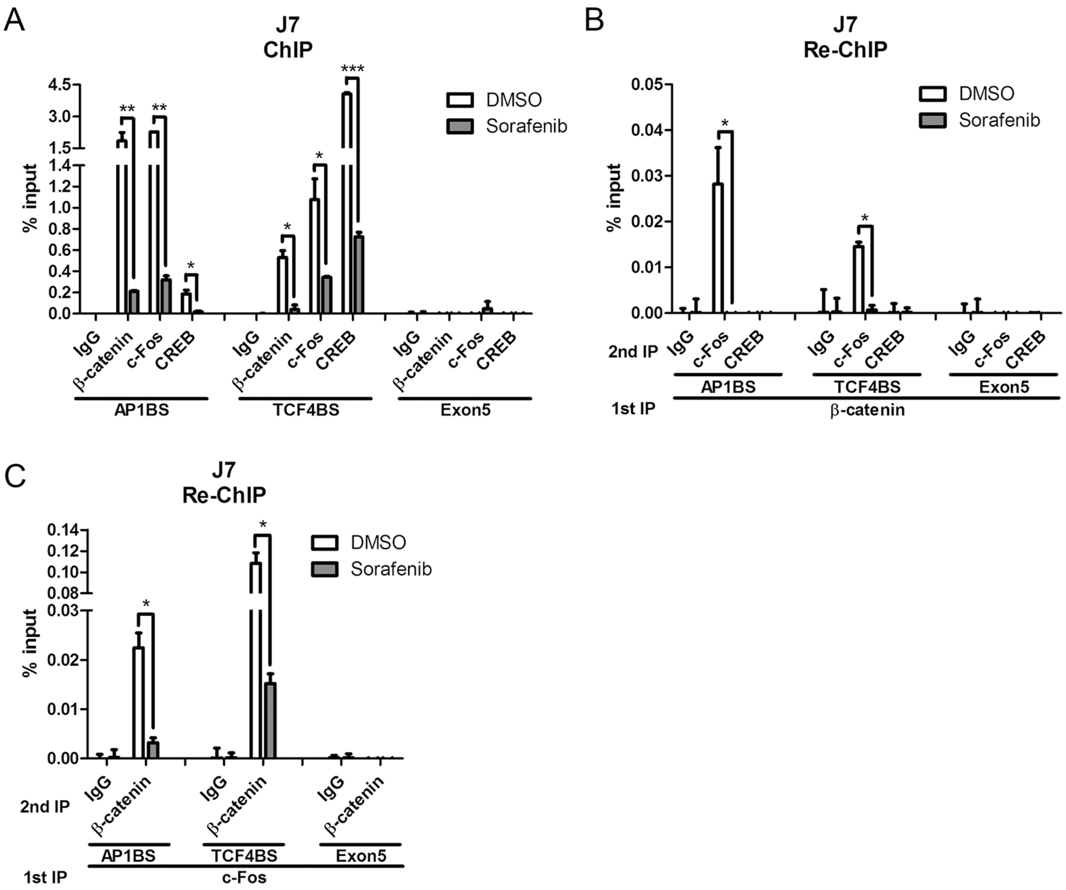

Supplementary Figure S3: Inhibition of β-catenin and associated c-Fos binding to PTMA promoter by sorafenib in J7 cells as assessed using ChIP assay. The results are the same as for Mahlavu cells (Figure 6).

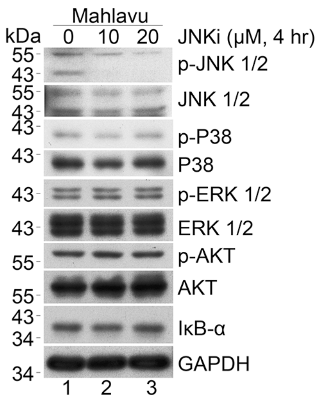

Supplementary Figure S4: Specific inhibition of JNK kinase activity by JNK inhibitor.

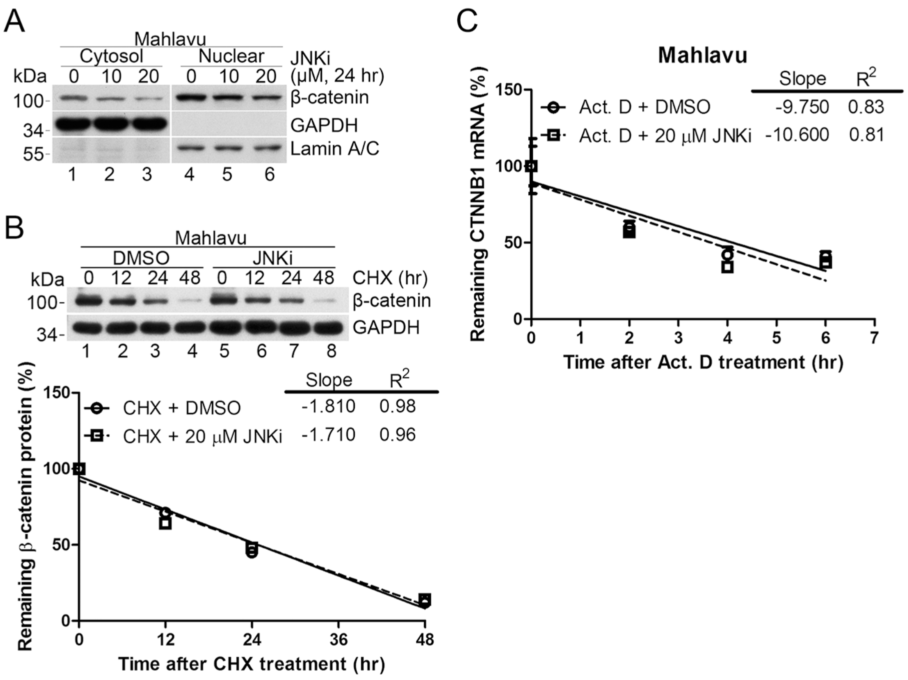

Supplementary Figure S5: Unaffected β-catenin protein and mRNA stability following treatment with JNK inhibitor. A. Reduction of β-catenin by JNK inhibitor in a dose-dependent manner. **B.** Degradation rate of β-catenin protein in Mahlavu cells treated with JNK inhibitor. Cells were pre-incubated with cycloheximide (CHX), followed by the indicated incubation. Linear regressions of the kinetic pattern of β-catenin protein level are shown at the bottom. **C.** Degradation rate of CTNNB1 mRNA in Mahlavu cells treated with JNK inhibitor. Cells were pre-incubated with actinomycin D (Act. D), followed by the indicated incubation. Results are shown as linear regression of the kinetic pattern of CTNNB1 mRNA level.

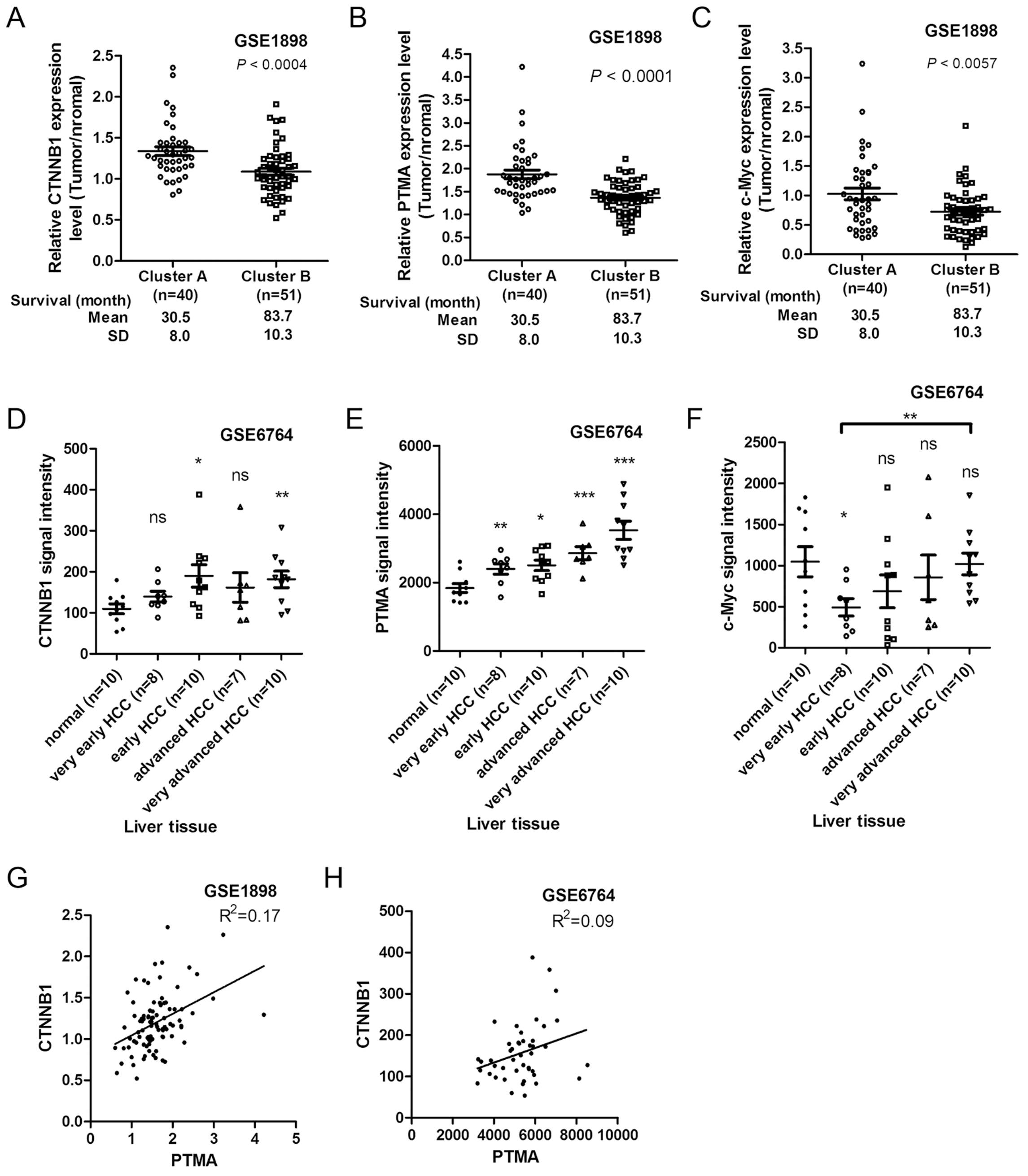

Supplementary Figure S6: Enhanced CTNNB1 and PTMA gene expression in human HCC patients. A–C. Positive correlation between high CTNNB1, PTMA and c-Myc gene expression status and poor prognosis of human HCC patients. Expression profile of microarray samples contains 91 human HCC patients from predefined subclasses as published by the National Cancer Institute/NIH (GEO accession: GSE1898). The significance of the difference between averages is indicated. **D–F** CTNNB1 and PTMA mRNA level is higher in patients with advanced HCC patients than in individuals with a healthy liver. c-Myc mRNA level is similar in these patients (F). Expression profile contains the indicated number of human HCC patients from predefined subclasses as published by the National Cancer Institute/NIH (GEO accession: GSE6764). **G, H.** Poor correlation between β-catenin and PTMA mRNA levels in clinical tumors. Correlation index was indicated.
